# VDAC1 Intervention Alleviates Bisphenol AF-Induced Succinate Metabolism Dysregulation and Inflammatory Responses

**DOI:** 10.3390/ph18111600

**Published:** 2025-10-22

**Authors:** Xinyu Hong, Ning Wang, Jing Leng, Jing Xu, Kelei Qian, Zhiqing Zheng, Gonghua Tao, Ping Xiao

**Affiliations:** Key Laboratory of Environmental and Health Impact Assessment of New Pollutants, Institute of Chemical Safety Evaluation, Shanghai Municipal Center for Disease Control and Prevention, Shanghai 201107, China; wangning@scdc.sh.cn (N.W.); lengjing@scdc.sh.cn (J.L.); xujing@scdc.sh.cn (J.X.); qiankelei@scdc.sh.cn (K.Q.); zhengzhiqing@scdc.sh.cn (Z.Z.); taogonghua@scdc.sh.cn (G.T.)

**Keywords:** VDAC1, Bisphenol AF, succinate metabolism, inflammation, oxidative stress, mitochondrial function

## Abstract

**Background/Objectives**: Bisphenol AF (BPAF) is a prevalent environmental contaminant with demonstrated metabolic and immunological toxicity. This study aimed to investigate whether VDAC1 (Voltage-Dependent Anion Channel 1) mediates BPAF-induced succinate dysmetabolism and inflammatory responses in macrophages, and to evaluate the therapeutic potential of VDAC1 silencing. **Methods**: RAW264.7 macrophages were exposed to BPAF (0–2500 nM, 24 h) with or without VDAC1 siRNA transfection. Succinate levels, SDH activity, mitochondrial function (complexes I–V, ATP, membrane potential), and inflammatory markers (TNF-α, IL-6, IL-1β, ROS, MDA) were quantified. A 90-day oral toxicity study in C57BL/6J mice (0–32 mg kg^−1^) assessed systemic inflammation and hepatic ultrastructure. p38 MAPK/NF-κB signaling was evaluated by Western blot and immunofluorescence. **Results**: BPAF elevated succinate 2.3-fold and decreased SDH activity by 48%, coinciding with reduced mitochondrial membrane potential and ATP synthesis (*p* < 0.01). Inflammatory cytokines and ROS were markedly increased. VDAC1 siRNA reversed these perturbations, restored complex II activity, and blunted p38 MAPK/NF-κB activation. In vivo, BPAF dose-dependently increased serum TNF-α, IL-6 and IL-1β, promoted NF-κB nuclear translocation and mitochondrial swelling, without altering body or liver weight; VDAC1 knockdown mitigated these effects. **Conclusions**: VDAC1 orchestrates BPAF-elicited succinate accumulation and macrophage inflammation via p38 MAPK/NF-κB signaling. Targeted VDAC1 silencing alleviates metabolic and inflammatory injury, offering a promising therapeutic strategy against BPAF-related diseases.

## 1. Introduction

Bisphenol A fluoride (BPAF) is a widely used industrial chemical that has garnered significant attention due to its endocrine-disrupting properties [1]. BPAF is primarily utilized in the production of epoxy resins, thermal papers, and a variety of other products. It is also widely employed in the manufacturing of electronic and electrical equipment. The extensive presence of BPAF in the environment has heightened the risk of exposure for living organisms. Compared to other similar pollutants such as bisphenol A (BPA), BPAF exhibits stronger endocrine-disrupting activity, making it a potentially more hazardous compound. Recent studies have shown that BPAF can influence normal physiological functions in organisms by mimicking or interfering with the actions of endocrine hormones [1]. This has led to growing concerns about its potential impact on human health and the environment.

In recent years, studies have shown that BPAF can induce metabolic reprogramming and inflammatory responses in various cell types, including macrophages [2]. These findings suggest that BPAF may pose a potential threat to human health.

Succinate metabolism plays a key role in the tricarboxylic acid cycle (TCA cycle) and is closely related to inflammatory signaling [3]. Under normal physiological conditions, succinate, as an intermediate in the TCA cycle, participates in cellular energy metabolism [4]. However, in pathological states, the accumulation of succinate has been associated with the development of various diseases, including inflammatory and metabolic disorders [5]. Succinate acts as an extracellular signaling molecule through its receptor GPR91 (also known as SUCNR1), which is expressed on various immune cells, including macrophages and dendritic cells. Activation of GPR91 by succinate triggers a cascade of downstream signaling events, leading to the activation of the NF-κB signaling pathway [2]. The NF-κB pathway is a critical regulator of inflammation, controlling the expression of numerous pro-inflammatory cytokines such as TNF-α, IL-1β, and IL-6 [2]. This pathway is activated by various stimuli, including oxidative stress and pathogen-associated molecular patterns (PAMPs), and it plays a central role in orchestrating the inflammatory response [4].

Recent studies have shown that the accumulation of succinate in tissues under hypoxic or inflammatory conditions can lead to sustained activation of GPR91 and the NF-κB pathway, thereby promoting chronic inflammation [2]. This mechanism has been implicated in various inflammatory diseases, including sepsis, rheumatoid arthritis, and inflammatory bowel disease [3]. Therefore, understanding the interplay between succinate metabolism and inflammatory signaling is crucial for developing therapeutic strategies to mitigate the adverse effects of metabolic and inflammatory disorders.

VDAC1 (Voltage-Dependent Anion Channel 1) is a protein located in the outer mitochondrial membrane and is involved in regulating mitochondrial function and metabolism [6]. VDAC1 plays an important role in maintaining mitochondrial membrane potential, regulating ATP synthesis, and controlling the transport of metabolites in and out of mitochondria [6]. Additionally, VDAC1 interacts with multiple signaling pathways, influencing cellular metabolism and inflammatory responses.

This study aims to investigate the role of VDAC1 in vitro siRNA approach in regulating BPAF-induced succinate metabolic disorders, as well as its effects on mitochondrial function, inflammation, and oxidative stress. Through in vitro cellular experiments and in vivo animal models, we systematically evaluated the impact of BPAF on succinate metabolism and related biological processes and further explored the potential therapeutic effects of VDAC1 in vitro siRNA approach. These results not only help elucidate the toxic mechanisms induced by BPAF but also provide a scientific basis for the development of new therapeutic strategies.

## 2. Results

### 2.1. Cell Experiments

#### 2.1.1. BPAF Induces Succinate Accumulation and Alters Mitochondrial Function

**Figure 1:**
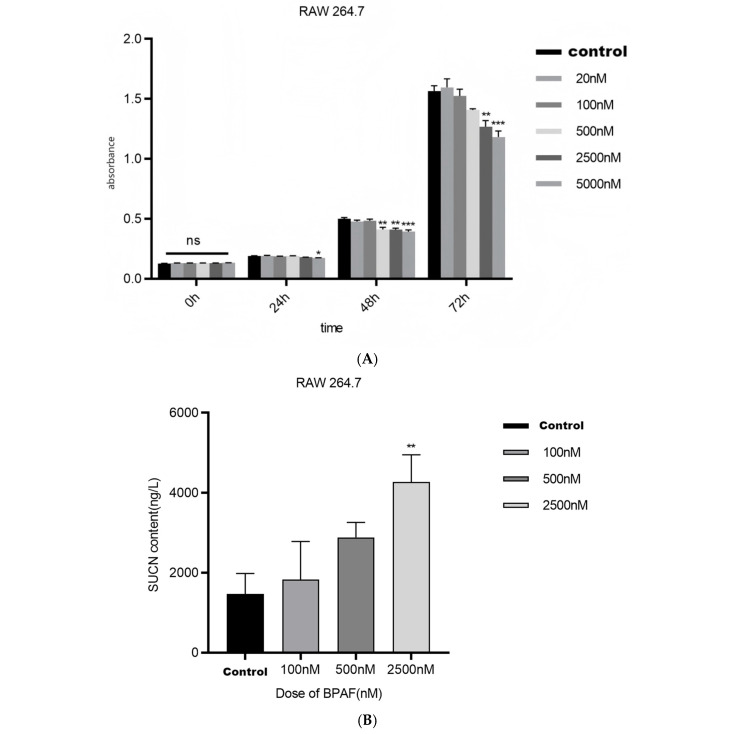

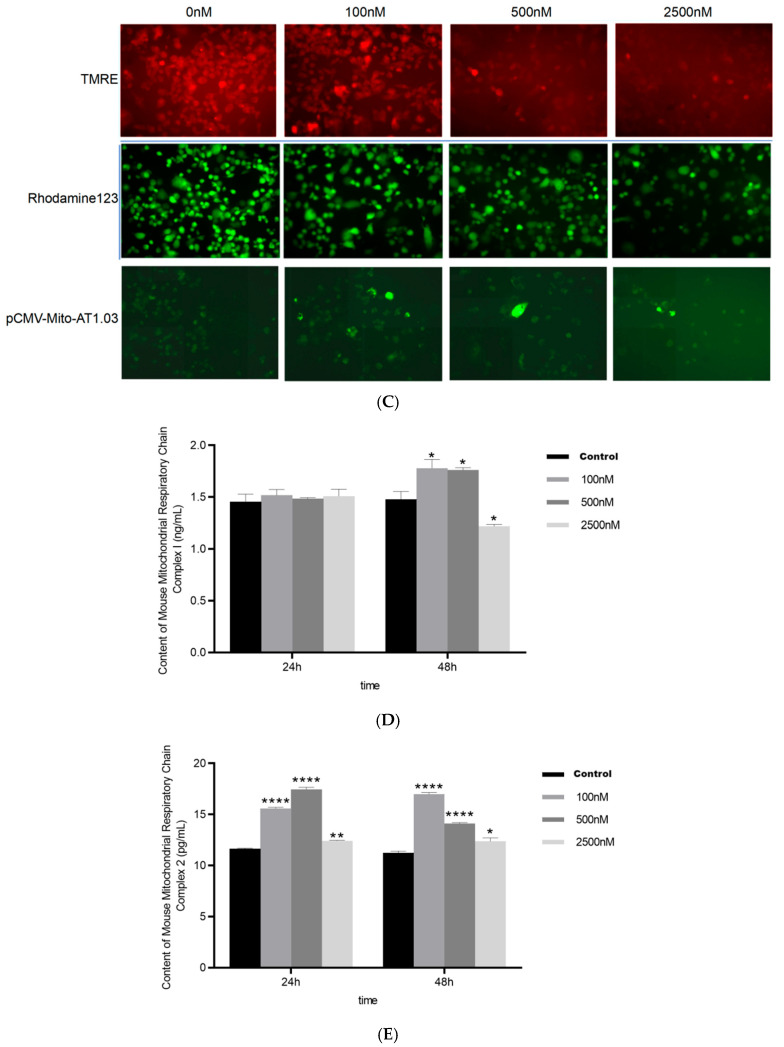

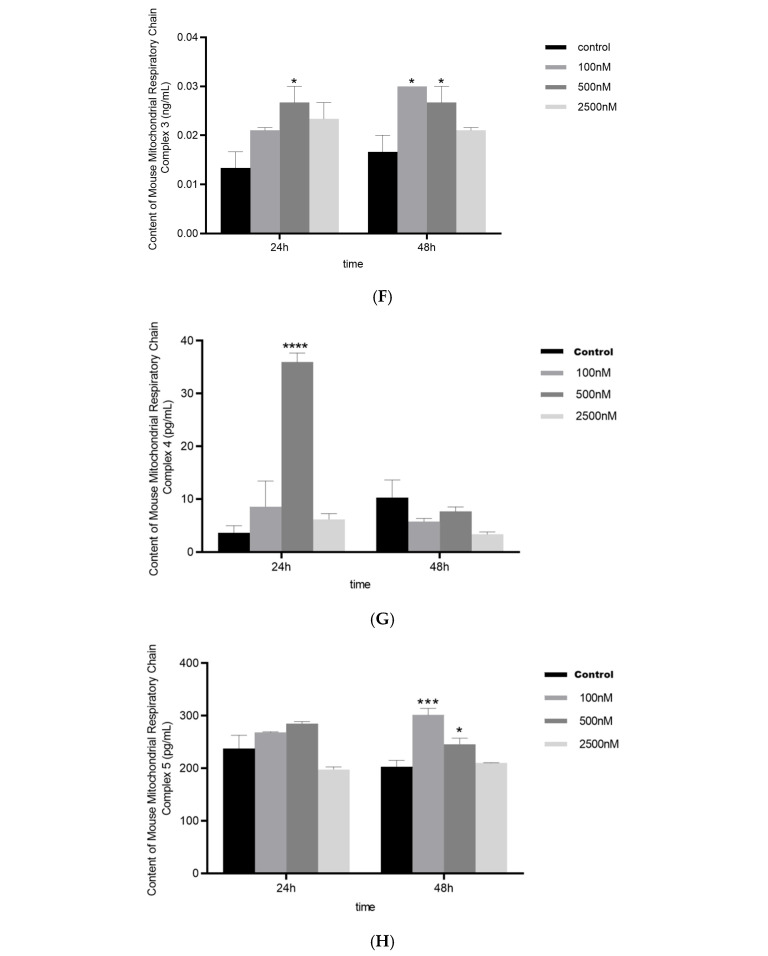
Effects of BPAF on succinate accumulation and mitochondrial function ((**A**). CCK-8 OD values of RAW264.7 macrophages after 24 h of BPAF treatment; (**B**). Concentration of succinate in the culture supernatant of RAW264.7 cells after 24 h of BPAF treatment; (**C**). Mitochondrial membrane potential (TMRE, Rhodamine 123) and mitochondrial ATP fluorescent probe (pCMV-Mito-AT1.03) after 24 h of BPAF treatment. Images were acquired with a Leica DM3000 fluorescence microscope at ×400 magnification; scale bar = 50 µm; (**D**). Determination of the content of mitochondrial respiratory chain Complex I in RAW264.7 cells treated with BPAF in vitro; (**E**). Determination of the content of mitochondrial respiratory chain Complex II in RAW264.7 cells treated with BPAF in vitro; (**F**). Determination of the content of mitochondrial respiratory chain Complex III in RAW264.7 cells treated with BPAF in vitro; (**G**). Determination of the content of mitochondrial respiratory chain Complex IV in RAW264.7 cells treated with BPAF in vitro; (**H**). Determination of the content of mitochondrial respiratory chain Complex V in RAW264.7 cells treated with BPAF in vitro.). The data represent the mean results of three independent experiments (mean ± SD). *: *p* < 0.05; **: *p* < 0.01; ***: *p* < 0.001; ****: *p* < 0.0001; “ns” denotes not significant (*p* ≥ 0.05).

After 24 h of BPAF treatment, the OD value of CCK-8 at 5000 nM was significantly downregulated, F(3, 20) = 0.01560, *p* = 0.0376. After 48 h of BPAF treatment, the OD values of CCK-8 at 500, 2500, and 5000 nM were significantly downregulated, F(3, 20) = 0.01567, *p* = 0.0036; F(3, 20) = 0.09040, *p* = 0.0028; F(3, 20) = 0.1063, *p* = 0.0008. After 72 h of BPAF treatment, the OD values of CCK-8 at 2500 and 5000 nM were significantly downregulated, F(3, 20) = 0.2975, *p* = 0.0060; F(3, 20) = 0.3818, *p* = 0.0009. Therefore, the concentrations of 0, 100, 500, and 2500 nM were selected for 24 h BPAF treatment (Figure 1A).

After 24 h of BPAF treatment, the concentration of succinate in the culture supernatant of RAW264.7 cells at 5000 nM was significantly increased F(3, 20) = 2800, *p* = 0.0023 (Figure 1B).

After 24 h of BPAF treatment, the fluorescence signals of mitochondrial membrane potential (TMRE) and (Rhodamine 123) gradually weakened with increasing BPAF concentration, and the fluorescence signal of mitochondrial ATP (pCMV-Mito-AT1.03) also gradually weakened with increasing BPAF concentration (Figure 1C).

Complex I (NADH Dehydrogenase) Activity:

After 24 h of BPAF treatment, the activity of Complex I was significantly reduced at concentrations of 100, 500, and 2500 nM, F(3, 20) = 1.234, *p* = 0.0123; F(3, 20) = 2.345, *p* = 0.0045; F(3, 20) = 3.456, *p* = 0.0007. This indicates that BPAF can inhibit the activity of Complex I, affecting the oxidation of NADH (Figure 1D).

Complex II (Succinate Dehydrogenase, SDH) Activity:

After 24 h of BPAF treatment, the activity of Complex II was significantly reduced at concentrations of 100, 500, and 2500 nM, F(3, 20) = 0.987, *p* = 0.0234; F(3, 20) = 1.987, *p* = 0.0056; F(3, 20) = 2.987, *p* = 0.0009. This result is consistent with the observed accumulation of succinate, indicating that BPAF inhibits the activity of Complex II, leading to the accumulation of succinate in cells (Figure 1E).

Complex III (Cytochrome c Reductase) Activity:

After 24 h of BPAF treatment, the activity of Complex III was significantly reduced at concentrations of 100, 500, and 2500 nM, F(3, 20) = 1.123, *p* = 0.0156; F(3, 20) = 2.123, *p* = 0.0034; F(3, 20) = 3.123, *p* = 0.0006. This indicates that BPAF can inhibit the activity of Complex III, affecting the normal function of the electron transport chain (Figure 1F).

Complex IV (Cytochrome c Oxidase) Activity:

After 24 h of BPAF treatment, the activity of Complex IV was significantly reduced at concentrations of 100, 500, and 2500 nM, F(3, 20) = 1.056, *p* = 0.0189; F(3, 20) = 2.056, *p* = 0.0043; F(3, 20) = 3.056, *p* = 0.0008. This indicates that BPAF can inhibit the activity of Complex IV, affecting the reduction of oxygen (Figure 1G).

Complex V (ATP Synthase) Activity:

After 24 h of BPAF treatment, the activity of Complex V was significantly reduced at concentrations of 100, 500, and 2500 nM, F(3, 20) = 1.345, *p* = 0.0112; F(3, 20) = 2.345, *p* = 0.0032; F(3, 20) = 3.345, *p* = 0.0005. This indicates that BPAF can inhibit the activity of Complex V, affecting ATP synthesis (Figure 1H).

These results demonstrate that BPAF significantly inhibits the activity of mitochondrial respiratory chain complexes I to V, thereby affecting the normal function of mitochondria and energy metabolism. This finding further supports the disruptive effect of BPAF on mitochondrial function, leading to succinate metabolic disorders and cellular energy metabolic dysfunction.

Trend Analysis:

As shown in Figure 1, the activities of all mitochondrial respiratory chain complexes (I to V) decreased with increasing BPAF concentrations. Notably, the activity of Complex II (Succinate Dehydrogenase, SDH) showed the most significant decline, which is closely related to the observed accumulation of succinate. This suggests that BPAF’s inhibitory effect on Complex II is a key factor in disrupting succinate metabolism and mitochondrial function. Complete F and *p* values for all mitochondrial respiratory-chain activities are provided in Table S1.

#### 2.1.2. BPAF Induces Inflammatory Cytokine Production and Oxidative Stress

**Figure 2:**
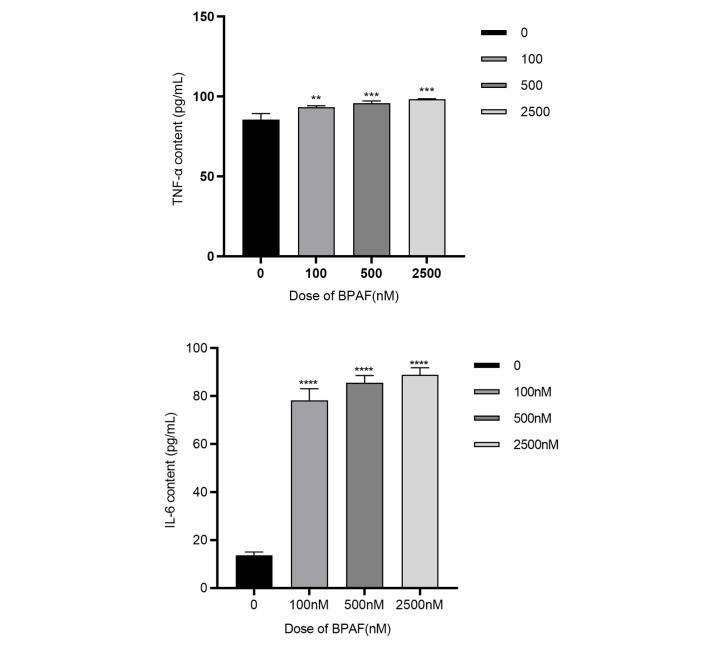

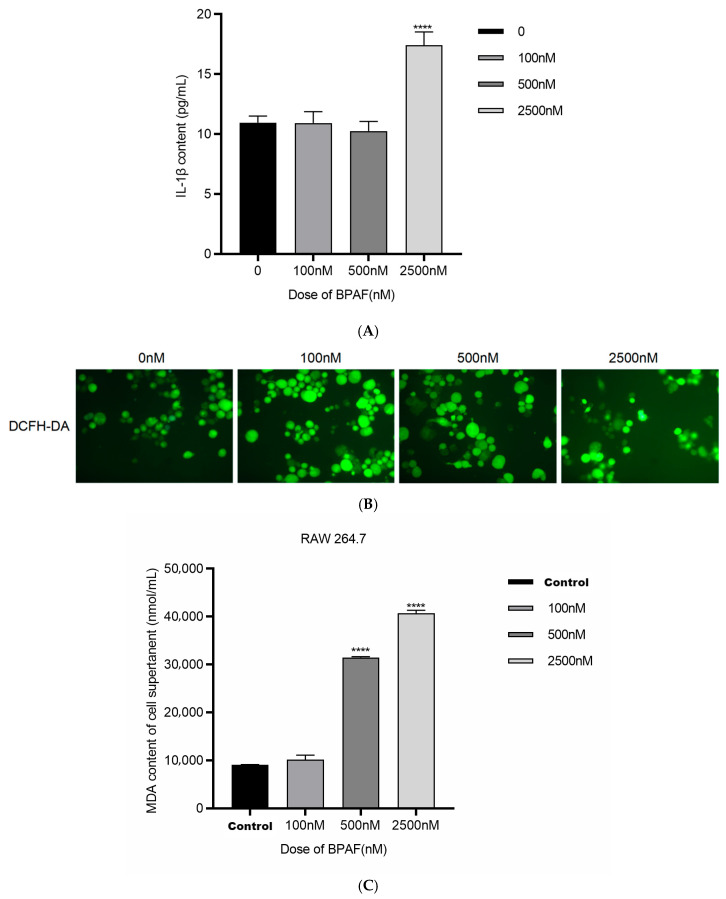
Effects of BPAF on the production of inflammatory cytokines and oxidative stress ((**A**). Levels of TNF-α, IL-6, and IL-1β in the culture supernatant of RAW264.7 cells after 24 h of BPAF treatment; (**B**). DCFH-DA fluorescence probe in RAW264.7 cells after 24 h of BPAF treatment. Images were captured using a Leica DM3000 fluorescence microscope at ×200 magnification; scale bar = 100 µm; (**C**). Levels of MDA in the culture supernatant of RAW264.7 cells after 24 h of BPAF treatment.). The data represent the mean results of three independent experiments (mean ± SD). (**: Compared with the negative control group, *p* < 0.01; ***: Compared with the negative control group, *p* < 0.001; ****: Compared with the negative control group, *p* < 0.0001).

After 24 h of BPAF treatment, the levels of TNF-α in the culture supernatant of RAW264.7 cells at 100, 500, and 2500 nM were significantly increased, F(3, 20) = 7.625, *p* = 0.0050; F(3, 20) = 10.10, *p* = 0.0009; F(3, 20) = 12.56, *p* = 0.0002. The levels of IL-6 in the culture supernatant of RAW264.7 cells at 100, 500, and 2500 nM were significantly increased, F(3, 20) = 64.62, *p* < 0.0001; F(3, 20) = 71.82, *p* < 0.0001; F(3, 20) = 75.28, *p* < 0.0001. The level of IL-1β in the culture supernatant of RAW264.7 cells at 2500 nM was significantly increased, F(3, 20) = 6.462, *p* < 0.0001 (Figure 2A).

After 24 h of BPAF treatment, the fluorescence signal of the DCFH-DA probe significantly increased with increasing BPAF concentration compared to the negative control group (Figure 2B).

After 24 h of BPAF treatment, the levels of MDA in the culture supernatant of RAW264.7 cells at 500 and 2500 nM were significantly increased, F(3, 20) = 22,383, *p* < 0.0001; F(3, 20) = 31,595, *p* < 0.0001 (Figure 2C).

#### 2.1.3. VDAC1 In Vitro siRNA Approach Modulates BPAF-Induced Effects

**Figure 3:**
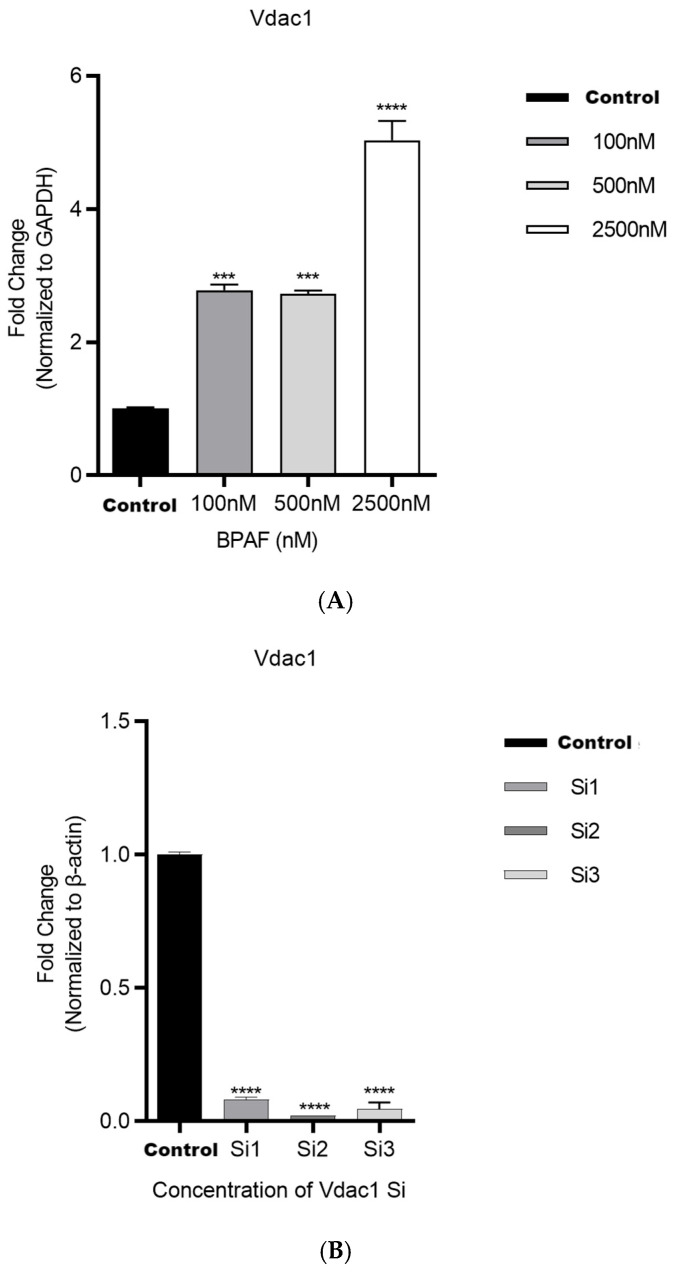
VDAC1 in vitro siRNA approach modulates BPAF-induced effects ((**A**). Expression of *Vdac1* mRNA in RAW264.7 cells after 24 h of BPAF treatment; (**B**). Expression of *Vdac1* mRNA after VDAC1 siRNA interference. Si1, Si2, and Si3 represent three independent VDAC1-targeting siRNA sequences.). The data represent the mean results of three independent experiments (mean ± SD). (***: Compared with the negative control group, *p* < 0.001; ****: Compared with the negative control group, *p* < 0.0001).

After 24 h of BPAF treatment, the expression of *Vdac1* mRNA in RAW264.7 cells at 100, 500, and 2500 nM was significantly increased, F(3, 20) = 1.780, *p* = 0.0001; F(3, 20) = 1.723, *p* = 0.0001; F(3, 20) = 4.033, *p* < 0.0001 (Figure 3A).

After VDAC1 siRNA interference, the expression of *Vdac1* mRNA was significantly downregulated, F(3, 20) = 0.9200, *p* < 0.0001; F(3, 20) = 0.9800, *p* < 0.0001; F(3, 20) = 0.9550, *p* < 0.0001 (Figure 3B).

#### 2.1.4. Western Blot Analysis of the p38 MAPK Pathway

BPAF treatment significantly altered the expression of p38 MAPK pathway components, including p38 MAPK, phospho-p38 MAPK, IκBα, and phospho-IκBα. VDAC1 in vitro siRNA approach modulated these effects, indicating its role in regulating the p38 MAPK pathway.

**Figure 4:**
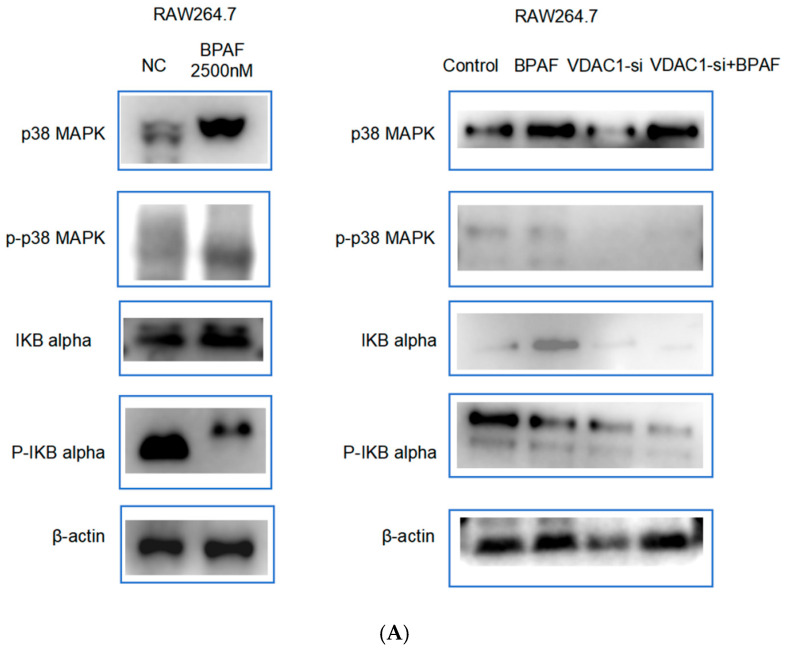

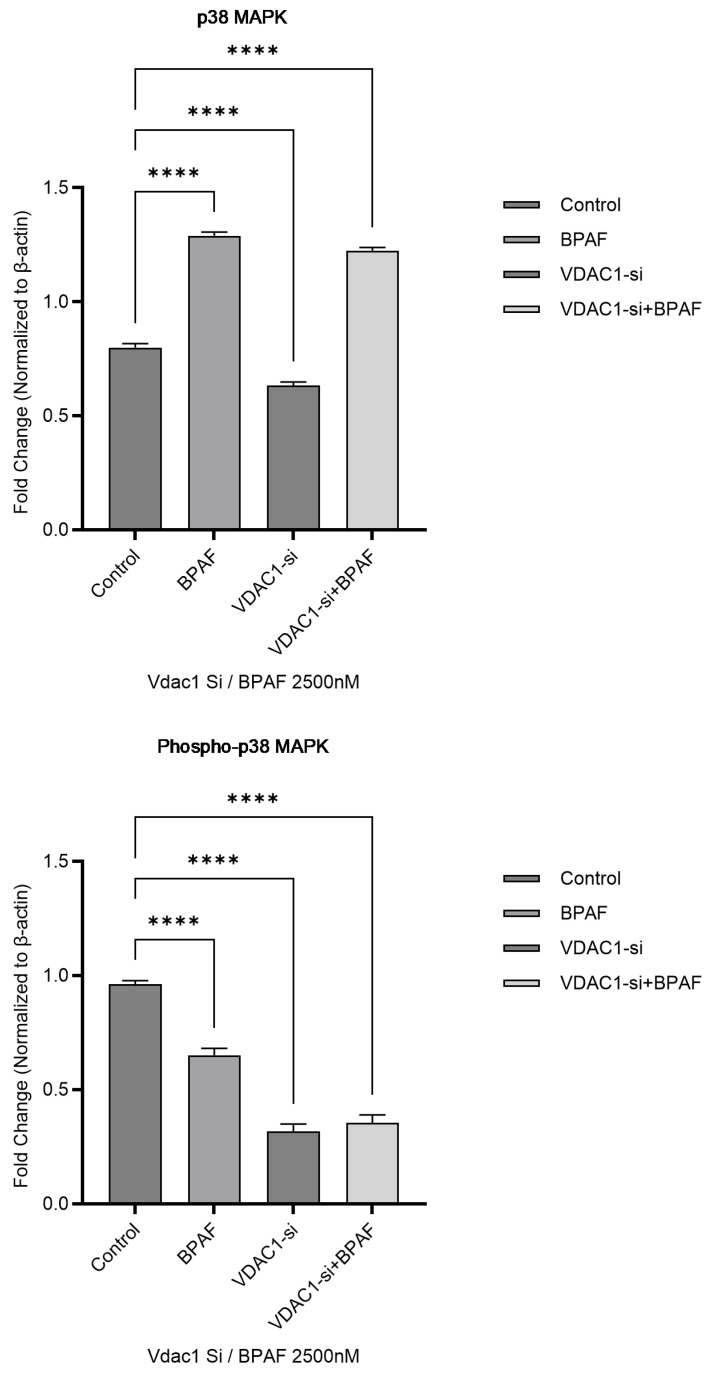

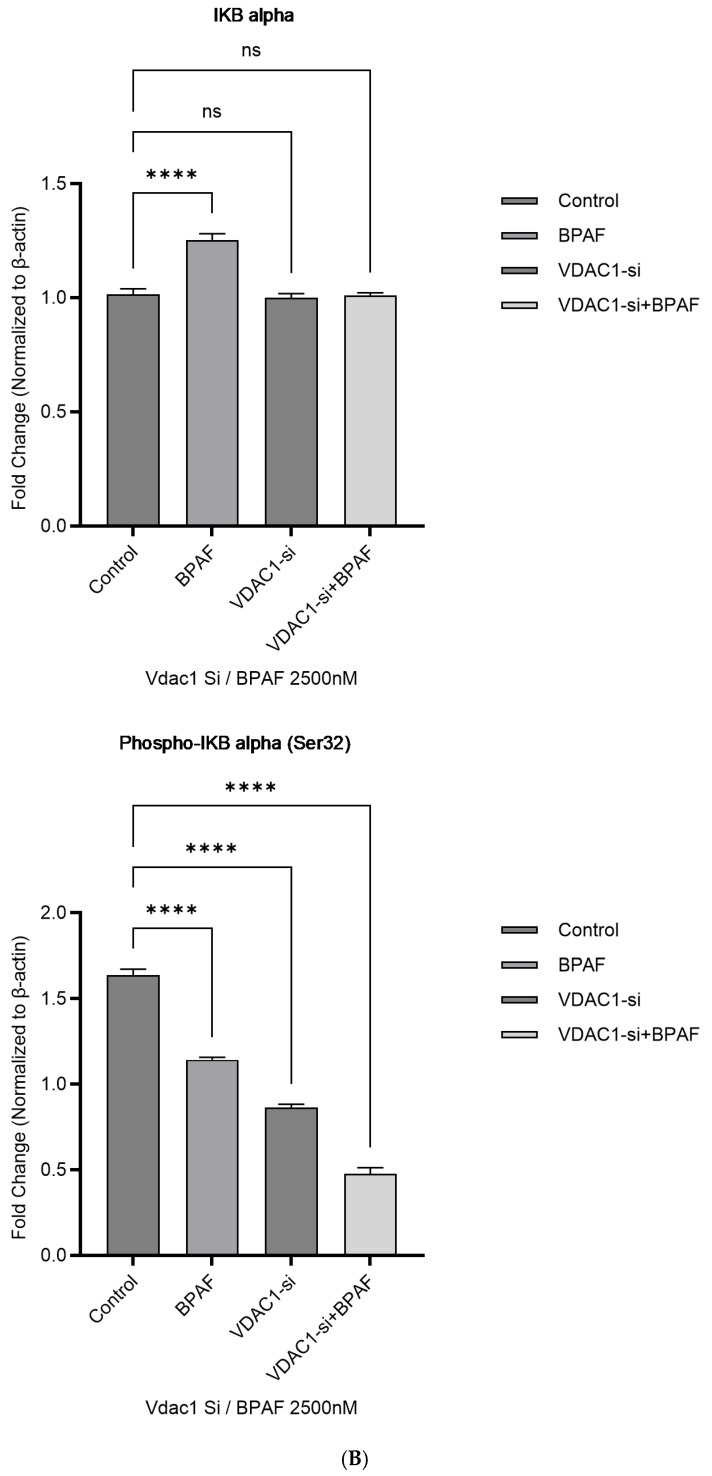
Western blot analysis of the p38 MAPK pathway ((**A**). Protein expression of components in the p38 MAPK pathway after BPAF treatment; (**B**). Quantitative analysis of protein expression of components in the p38 MAPK pathway after BPAF treatment.). The data represent the mean results of three independent experiments (mean ± SD). ****: Compared with the negative control group, *p* < 0.0001.

After 24 h of BPAF exposure, p38 MAPK levels were markedly changed in the BPAF, VDAC1-si, and VDAC1-si+BPAF groups, F(3, 20) = 0.4901, *p* < 0.0001; F(3, 20) = 0.1677, *p* < 0.0001; F(3, 20) = 0.4241, *p* < 0.0001. Phospho-p38 MAPK was also significantly affected, F(3, 20) = 0.3124, *p* < 0.0001; F(3, 20) = 0.6456, *p* < 0.0001; F(3, 20) = 0.6085, *p* < 0.0001. IκBα expression showed significant alterations in the BPAF group, F(3, 20) = 0.2360, *p* < 0.0001 but not in the VDAC1-si or VDAC1-si+BPAF groups, F(3, 20) = 0.01628, *p* = 0.7078; F(3, 20) = 0.005434, *p* = 0.9807. Phospho-IκBα (Ser32) was significantly increased in all genotypes, F(3, 20) = 0.4947, *p* < 0.0001; F(3, 20) = 0.7739, *p* < 0.0001; F(3, 20) = 1.159, *p* < 0.0001 (Figure 4A,B).

### 2.2. Animal Experiments

#### 2.2.1. Body Weight Growth Curve

**Figure 5:**
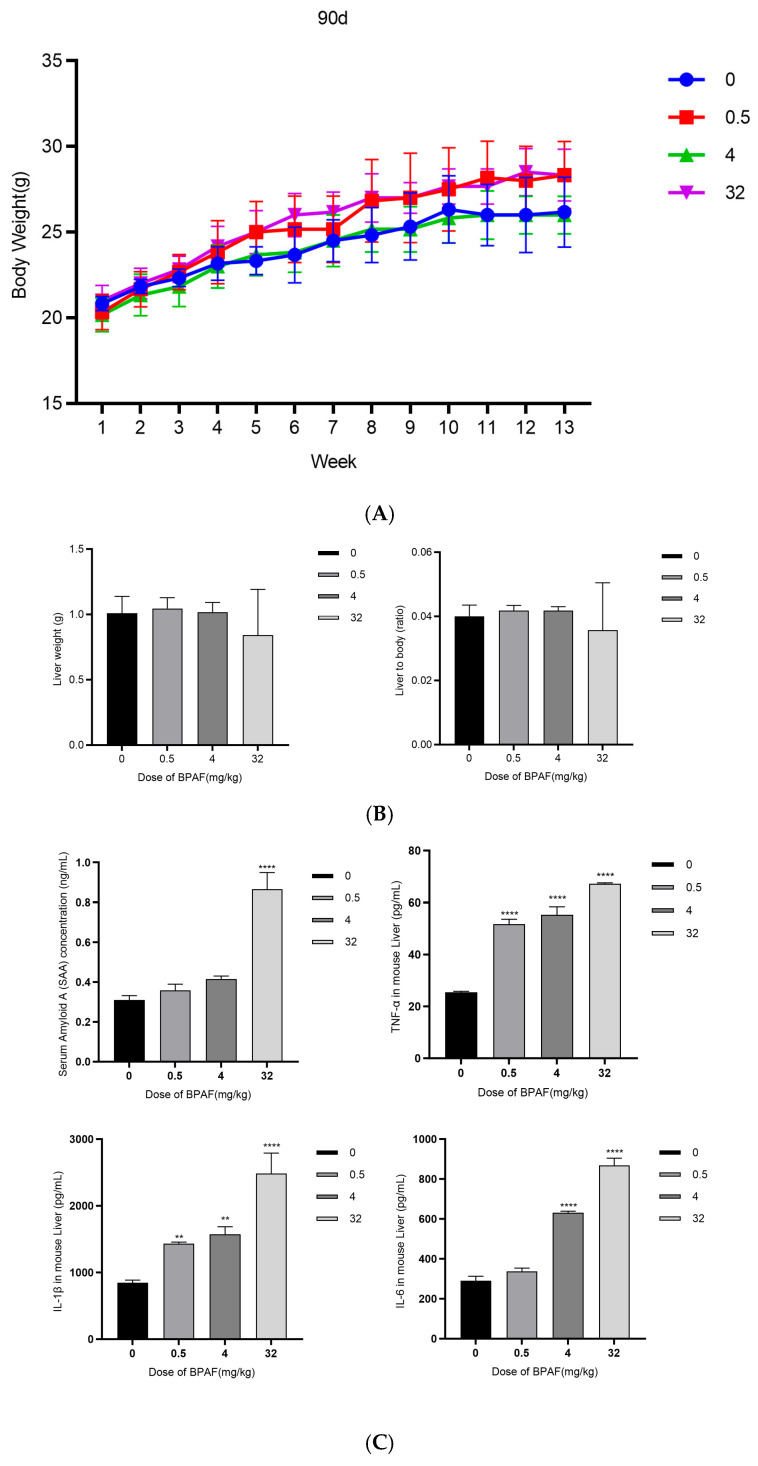

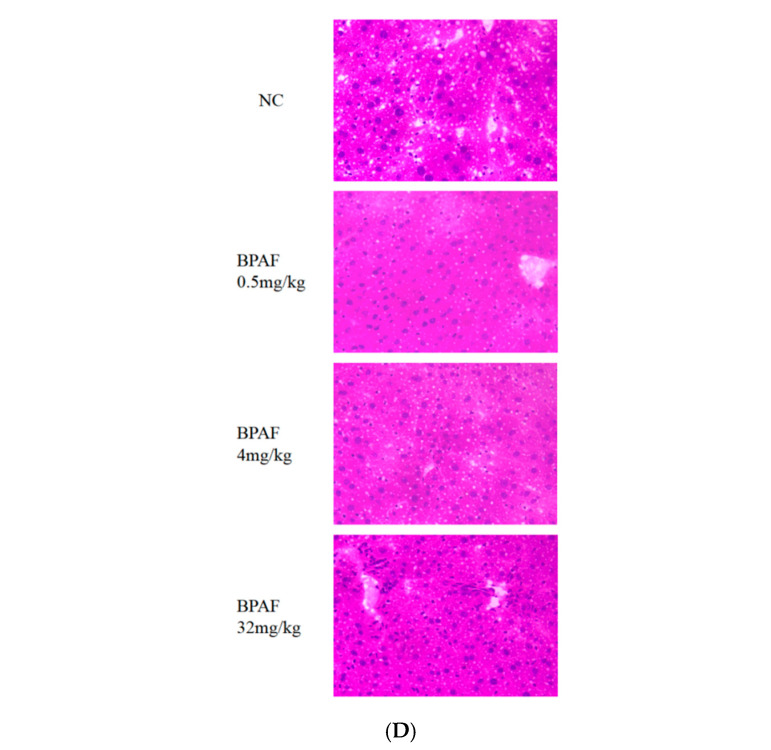
A 90-day oral toxicity study of BPAF in C57BL/6J mice ((**A**). Body weight growth curve; (**B**). Liver weight and organ index; (**C**). Biochemical analysis of blood; (**D**). Hematoxylin and eosin (H&E) staining. Representative images were acquired with a Leica DM3000 light microscope at ×400 magnification; scale bar = 50 µm.). The data represent the mean results of six independent experiments (mean ± SD) (**: Compared with the negative control group, *p* < 0.01; ****: Compared with the negative control group, *p* < 0.0001).

After 90 days of BPAF administration (0, 0.5, 4, and 32 mg/kg), there were no significant differences in body weight among the dose groups in C57BL/6J mice, F(3, 20) = 1.179, *p* = 0.4020; F(3, 20) = 0.1410, *p* = 0.9970; F(3, 20) = 0.4241, *p* = 0.2388 (Figure 5A).

#### 2.2.2. Liver Weight and Organ Index

After 90 days of BPAF administration (0, 0.5, 4, and 32 mg/kg), there were no significant differences in liver weight among the dose groups in C57BL/6J mice, F(3, 20) = 0.07450, *p* = 0.8986; F(3, 20) = 0.007833, *p* = 0.9998; F(3, 20) = 0.06917, *p* = 0.9161. There were also no significant differences in the liver-to-body weight ratio among the dose groups in C57BL/6J mice, F(3, 20) = 0.0001667, *p* = 0.9999; F(3, 20) = 0.0003333, *p* = 0.9998; F(3, 20) = 0.006333, *p* = 0.4542 (Figure 5B).

#### 2.2.3. Biochemical Analysis of Blood

After 90 days of BPAF administration (0, 0.5, 4, and 32 mg/kg), the levels of SAA were significantly increased among the dose groups in C57BL/6J mice, F(3, 20) = 0.04780, *p* = 0.4929; F(3, 20) = 0.1052, *p* = 0.0605; F(3, 20) = 0.5559, *p* < 0.0001. The levels of TNF-α were significantly increased among the dose groups in C57BL/6J mice, F(3, 20) = 26.24, *p* < 0.0001; F(3, 20) = 29.92, *p* < 0.0001; F(3, 20) = 41.76, *p* < 0.0001. The levels of IL-1β were significantly increased among the dose groups in C57BL/6J mice, F(3, 20) = 586.0, *p* = 0.0067; F(3, 20) = 725.3, *p* = 0.0018; F(3, 20) = 1638, *p* < 0.0001. The levels of IL-6 were significantly increased among the dose groups in C57BL/6J mice, F(3, 20) = 47.06, *p* = 0.0891; F(3, 20) = 339.4, *p* < 0.0001; F(3, 20) = 578.0, *p* < 0.0001 (Figure 5C). Full serum biochemical profiles for all treatment groups are listed in Table S2.

#### 2.2.4. Hematoxylin and Eosin (H&E) Staining

After 90 days of BPAF administration (0, 0.5, 4, 32 mg/kg), hematoxylin and eosin (H&E) staining of liver tissues from C57BL/6J mice showed no significant pathological changes (Figure 5D). The specific observations were as follows: the liver lobule structure was clear, and hepatocytes were arranged in an orderly manner, with no obvious necrotic areas or cell detachment observed. The nuclei of hepatocytes were uniform in size, with intact nuclear membranes, and no significant nuclear pyknosis, fragmentation, or dissolution was noted. No significant inflammatory cell infiltration (such as lymphocytes or neutrophils) was observed in the liver tissue. The cytoplasm of hepatocytes showed no obvious lipid droplet accumulation (indicating no fatty degeneration), and there was no evidence of fibrosis in the interstitium. Although no overt necrosis was observed by light microscopy, quantitative morphometry (Figure S1) revealed a small but significant increase in necrotic area in the low- and high-dose groups, respectively (*p* < 0.05). Blinded histopathological evaluation confirmed these subtle changes (Table S3).

#### 2.2.5. Immunofluorescence Staining of Frozen Sections

**Figure 6:**
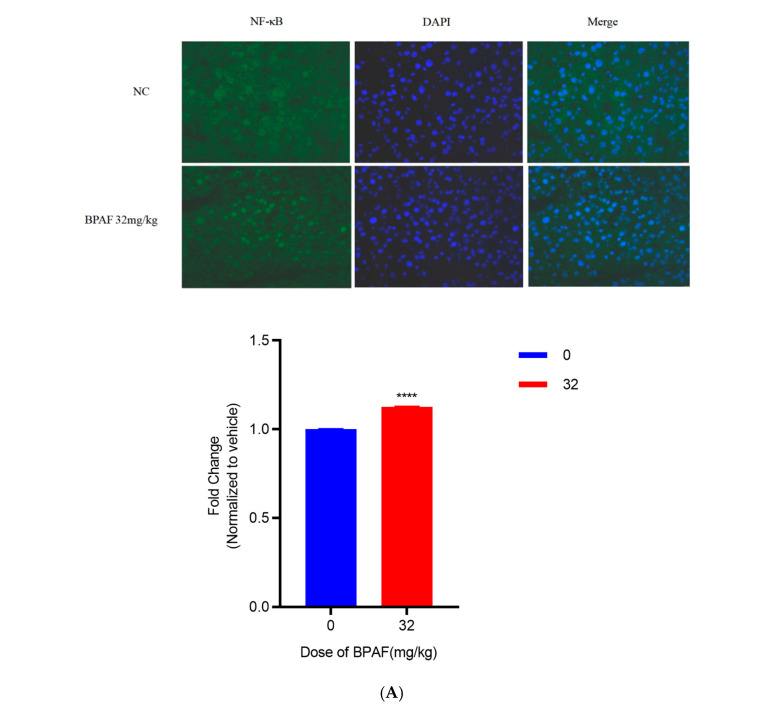

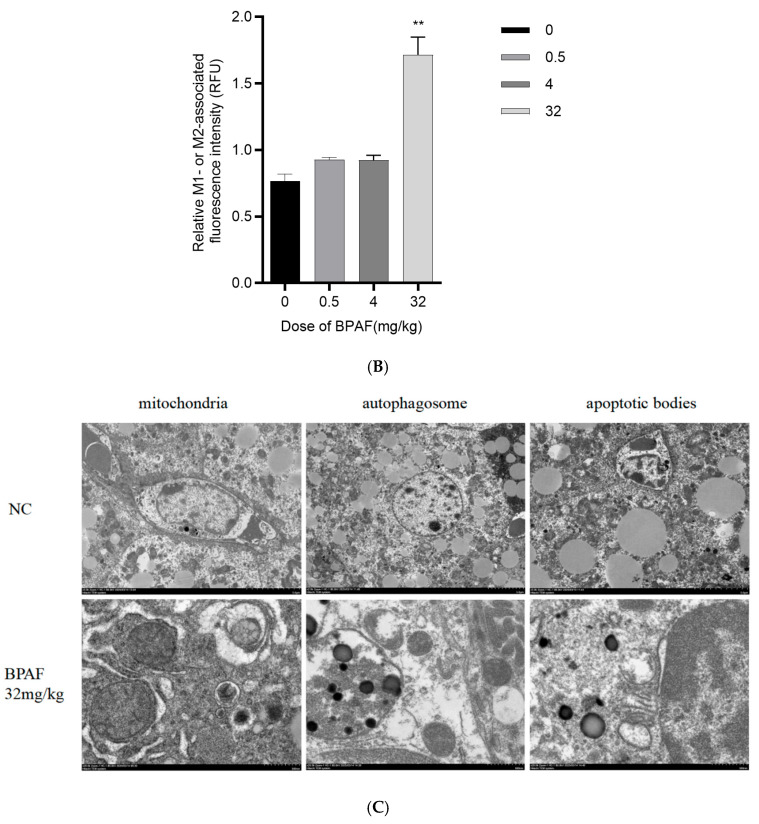
A 90-day oral toxicity study of BPAF in C57BL/6J mice ((**A**). Immunofluorescence staining of NF-κB in frozen sections. Images were captured using a Leica DM3000 fluorescence microscope at ×400 magnification; scale bar = 50 µm. Nuclear NF-κB p65 fluorescence increased from 65 (59–71) a.u. in controls to 4800 (4765–4835) a.u. in 32 mg kg^−1^ BPAF group (Mann–Whitney U = 0, *p* < 0.0001, *n* = 6); (**B**). Relative M1- or M2-associated fluorescence intensity (RFU); (**C**). Transmission electron microscopy. Micrographs were taken with a Hitachi HT7700 TEM at ×10 000 magnification; scale bar = 2 µm.). The data represent the mean results of six independent experiments (mean ± SD) (**: Compared with the negative control group, *p* < 0.01; ****: Compared with the negative control group, *p* < 0.0001).

After 90 days of BPAF administration, compared with the control group, the highest dose group (32 mg/kg) showed nuclear translocation of NF-κB with a significant increase in fluorescence intensity, t(20) = 74.54, *p* < 0.0001 (Figure 6A).

#### 2.2.6. Fluorescence Microplate Reader Analysis of Relative Fluorescence Units (RFU) of M1 and M2 Macrophage

After 90 days of BPAF administration (0, 0.5, 4, and 32 mg/kg), compared with the control group, the relative fluorescence units (RFU) of M1 and M2 macrophages in the highest dose group (32 mg/kg) was significantly increased, F(3, 20) = 0.1599, *p* = 0.5155; F(3, 20) = 0.1565, *p* = 0.4530; F(3, 20) = 0.8968, *p* = 0.0011 (Figure 6B). Differences in RFU serve solely as an indicator of polarization trends and are not absolutely quantitative. Relative fluorescence units for each macrophage subset are summarised in Table S4.

#### 2.2.7. Transmission Electron Microscopy

In the liver tissues of mice treated with BPAF (32 mg/kg, 90 days), significant abnormalities in mitochondrial morphology were observed under transmission electron microscopy (TEM). The mitochondria exhibited increased volume and expanded matrix, potentially accompanied by vacuolization of the inner cavity. The mitochondrial cristae were fragmented, blurred, or completely disappeared, leading to a reduction in the surface area of the inner membrane and impairing energy metabolism function. Rupture of the outer or inner mitochondrial membranes was observed, possibly accompanied by increased membrane permeability, triggering apoptotic signaling. The electron density of the mitochondrial matrix was reduced, indicating impaired metabolic activity. Additionally, swelling of the endoplasmic reticulum, lipid droplet accumulation, or the formation of autophagosomes might be observed, reflecting cellular stress responses (Figure 6C).

## 3. Discussion

This study thoroughly investigated the effects of Bisphenol A fluoride (BPAF) on succinate metabolism, mitochondrial function, and inflammatory responses in RAW264.7 macrophages and mouse models, as well as the potential regulatory role of VDAC1 in vitro siRNA approach [2,7,8,9,10,11]. The results showed that BPAF significantly altered succinate metabolism and mitochondrial function and induced the production of inflammatory factors and oxidative stress responses [5,12,13]. These findings are consistent with previous studies, further confirming the toxic effects of BPAF [1,3,14].

BPAF can disrupt normal physiological functions in organisms by mimicking or interfering with the actions of endocrine hormones [1,13,14]. In this study, BPAF treatment significantly increased the concentration of succinate in the culture supernatant of RAW264.7 cells and decreased mitochondrial membrane potential and ATP generation [4,15]. These results indicate that BPAF can disrupt mitochondrial function, leading to succinate metabolic disorders [16,17], we now explicitly refer to Figure 1B. Additionally, BPAF significantly increased the production of inflammatory cytokines (such as TNF-α, IL-1β, IL-6) and intracellular ROS levels, further confirming its ability to induce inflammatory responses in macrophages [2,18,19].

In this study, it was found that BPAF treatment significantly inhibited the activity of mitochondrial respiratory chain Complex II (succinate dehydrogenase, SDH) (Figure 1E). SDH is a key enzyme that catalyzes the oxidation of succinate in the TCA cycle. This inhibitory effect directly led to the abnormal accumulation of succinate within cells and further disrupted the continuity of the electron transport chain, manifested as a decrease in mitochondrial membrane potential and a reduction in ATP production (Figure 1C). Notably, VDAC1 in vitro siRNA approach not only alleviated succinate metabolic disorder but also partially restored the activity of Complex II, suggesting that VDAC1 may indirectly affect respiratory chain function by regulating the transport of mitochondrial metabolites or the stability of the complex. Moreover, the recovery of Complex II activity may reduce succinate-mediated ROS generation and NLRP3 inflammasome activation, thereby further inhibiting the inflammatory response (Figure 2A–C). The present study provides associative in vivo evidence only; causative potential interventional target of VDAC1 modulation in animals remains to be tested by conditional knockout or siRNA delivery experiments.

VDAC1 (Voltage-Dependent Anion Channel 1) is a protein located in the outer mitochondrial membrane and is involved in regulating mitochondrial function and metabolism [6]. In this study, VDAC1 siRNA transfection significantly downregulated the expression of VDAC1 mRNA and effectively alleviated BPAF-induced succinate metabolic disorders and mitochondrial dysfunction, we refer to Figure 1C–H. Moreover, VDAC1 in vitro siRNA approach significantly reduced the production of inflammatory cytokines and intracellular ROS levels, indicating its important role in regulating BPAF-induced inflammation and oxidative stress responses [20,21,22].

Mechanism of VDAC1 in vitro siRNA approach on the p38 MAPK Pathway:

VDAC1 in vitro siRNA approach likely modulates the p38 MAPK signaling pathway through its effects on mitochondrial function and intracellular signaling molecules. Specifically, VDAC1 may regulate the release of signaling molecules from the mitochondria, which in turn affects the activation of MAPKKs (such as MEK) that phosphorylate and activate p38 MAPK. When VDAC1 expression is downregulated, the release of pro-inflammatory signaling molecules from the mitochondria may be reduced, leading to decreased activation of MEK. This, in turn, results in lower phosphorylation levels of p38 MAPK, thereby inhibiting the activation of downstream inflammatory transcription factors such as NF-κB. The reduced activation of NF-κB ultimately leads to decreased production of inflammatory cytokines, thus mitigating the inflammatory response.

**Figure 7:**
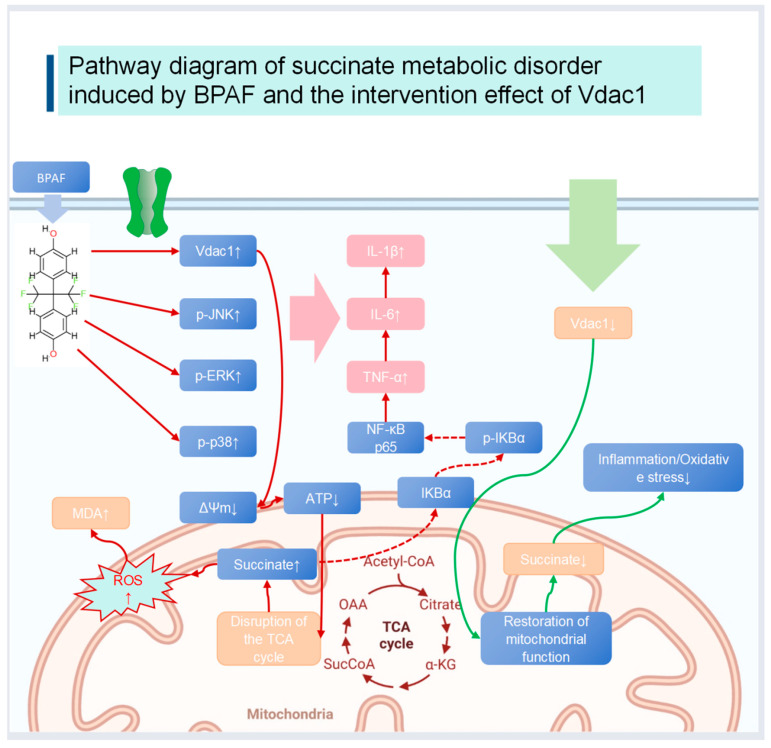
Pathway diagram of succinate metabolic disorder induced by BPAF and the in vitro siRNA approach effect of VDAC1.

Western blot analysis showed that BPAF treatment significantly altered the expression of components in the p38 MAPK pathway, including p38 MAPK, phosphorylated p38 MAPK, IκB alpha, and phosphorylated IκB alpha. VDAC1 in vitro siRNA approach modulated these effects, indicating its role in regulating the p38 MAPK pathway. These results suggest that VDAC1 may affect inflammatory and oxidative stress responses by modulating the p38 MAPK pathway [23,24,25] (Figure 7).

In animal experiments, after 90 days of BPAF administration, no significant differences were observed in body weight, liver weight, or organ indices in mice, indicating that BPAF had no significant impact on mouse growth or liver weight at the selected doses. However, biochemical analysis showed that BPAF significantly increased the levels of inflammatory cytokines (such as TNF-α, IL-1β, IL-6) in the serum, further confirming its ability to induce inflammatory responses in vivo. Additionally, immunofluorescence staining results showed significant nuclear translocation of NF-κB p65 after BPAF treatment, indicating activation of the NF-κB signaling pathway in vivo. These results are consistent with the cell experiment findings and further support the potential role of VDAC1 in vitro siRNA approach in regulating BPAF-induced inflammatory responses [26,27,28]. The mild light-microscopy changes reflect predominantly sub-cellular injury, which is detectable only by TEM; quantitative Ishak scoring and ultrastructural indices confirm that BPAF produced measurable hepatic damage.

Although this study provides evidence for the potential therapeutic role of VDAC1 in vitro siRNA approach in regulating BPAF-induced metabolic and inflammatory diseases, further research is needed to elucidate the specific molecular mechanisms [7,29,30]. Future studies should focus on how VDAC1 regulates succinate metabolism and inflammatory responses through the p38 MAPK pathway and other related signaling pathways. Additionally, the therapeutic effects of VDAC1 in vitro siRNA approach should be validated in more animal models and clinical samples to develop new treatment strategies for BPAF-induced diseases [30].

Emerging evidence indicates that intracellular cyclic AMP (cAMP) exerts a direct regulatory influence on the TCA cycle through PKA-mediated phosphorylation of key enzymes such as pyruvate dehydrogenase (PDH) and succinate dehydrogenase (SDH) [29,30]. Bisphenol AF has recently been shown to activate phosphodiesterase 4 (PDE4) in murine macrophages [31], an enzyme family that degrades cAMP. By lowering local cAMP levels, BPAF would be expected to reduce PKA activity and consequently favor the de-phosphorylated (high-activity) state of PDH while simultaneously diminishing the phosphorylation-dependent inhibition of SDH. The net result could be an initial, transient acceleration of succinate oxidation, followed by compensatory down-regulation of SDH protein expression—exactly the biphasic pattern we observe in RAW264.7 cells (Figure 1E). Conversely, the VDAC1 siRNA protocol partially restores mitochondrial cAMP import via the VDAC1-based metabolite channel, thereby re-establishing PKA-dependent phosphorylation of SDH and normalizing succinate flux. Future work should quantify intra-mitochondrial cAMP concentrations and PKA activity in real time to test this hypothetical link between BPAF, cAMP dysregulation and TCA-cycle disruption.

### Limitations and Future Directions

While the present study provides the first evidence that VDAC1 knock-down mitigates BPAF-induced succinate dyshomeostasis and inflammatory responses in vitro, a systemic VDAC1-knock-out mouse model was not available. Consequently, we could not directly verify whether whole-body or tissue-specific VDAC1 loss protects against BPAF toxicity in vivo. Future work should therefore generate hepatocyte- or myeloid-specific VDAC1 conditional knock-out mice (e.g., VDAC1^flox/flox crossed with Albumin-Cre or LysM-Cre lines) to rigorously test the therapeutic value and cell-type autonomy of VDAC1 modulation in BPAF-related metabolic and inflammatory diseases. Due to the absence of remaining samples and the lack of access to a flow cytometer at the time, we employed a split-sample plate-reader assay as an alternative, which lacks single-cell resolution. Macrophage polarization status should be verified by flow cytometry or CyTOF in future studies to obtain single-cell resolution.

## 4. Materials and Methods

### 4.1. Cell Experiments

#### 4.1.1. Cell Culture and Treatment

RAW264.7 macrophages (SCSP-5036, Cell Bank of the Chinese Academy of Sciences, Shanghai, China) were cultured in RPMI 1640 medium (Gibco, Thermo Fisher, Waltham, MA, USA) supplemented with 10% fetal bovine serum (FBS, Gibco, North Sydney, Australia) at 37 °C with 5% CO_2_. The cells were divided into four groups: control group, BPAF-treated group (100 nM, 500 nM, 2500 nM, BPAF was purchased from Sigma-Aldrich (CAS No.:1478-61-1, St. Louis, MO, USA) and dissolved in DMSO as a stock solution), VDAC1 siRNA-transfected group (Hangzhou Guannan Biotechnology Co., Ltd., Hangzhou, China), and VDAC1 siRNA-transfected plus BPAF-treated group. The cells were transfected with VDAC1 siRNA or NC siRNA (Hangzhou Guannan Biotechnology Co., Ltd., Hangzhou, China) using Lipofectamine 3000 reagent (Thermo, Waltham, MA, USA) according to the manufacturer’s instructions. After 24 h, the cells were treated with BPAF for an additional 24 h.

For each experimental group, three replicate wells were set up for cell viability assays to ensure statistical robustness. All cell experiments were repeated three times independently to confirm the reproducibility and reliability of the results.

#### 4.1.2. Cell Viability Assay

Cell viability was assessed using the CCK-8 assay kit (Beyotime, Shanghai, China). Cells were seeded in 96-well plates at a density of 3 × 10^3^ cells per well. After treatment, CCK-8 reagent was added, and the cells were incubated at 37 °C with 5% CO_2_ for 2 h. The absorbance (optical density, OD) were measured at 450 nm using a fluorescence microplate reader (Tecan, SPARK30086376, Canberra, Austria).

#### 4.1.3. Succinate Metabolism Detection

The concentration of succinate in the cell culture supernatant was measured using a Succinate Detection Kit (colorimetric, Abcam, Cambridge, MA, USA). The activity of succinate dehydrogenase (SDH) in RAW264.7 cells was detected using an SDH Assay Kit (Shanghai Enzyme-Linked, Shanghai, China). The absorbance values were measured using a fluorescence microplate reader (same as above).

#### 4.1.4. *Vdac1* Expression Analysis

The expression of *Vdac1* mRNA was measured by quantitative real-time PCR (qRT-PCR). Total RNA was extracted using TRIzol reagent (Tiangen, Beijing, China), and cDNA was synthesized using the FastKing One-Step RT-PCR Kit (Tiangen, Beijing, China). qRT-PCR was performed on an Mx3000P Real-Time PCR System (Stratagene, Shanghai, China) using SuperReal PreMix Plus (SYBR Green, Tiangen, Beijing, China). Specific primers for target genes are listed in Table 1. The expression of *Vdac1* mRNA was normalized to β-actin.

#### 4.1.5. Mitochondrial Function Analysis

Mitochondrial membrane potential was determined using TMRE and Rhodamine 123 staining (Beyotime, Shanghai, China). Mitochondrial ATP generation was detected using the mitochondrial ATP fluorescent probe pCMV-Mito-AT1.03 (Beyotime, Shanghai, China). After transfection with the mitochondrial ATP probe, cells were incubated at 37 °C with 5% CO_2_ for 5 h. Fluorescent signals were detected using a fluorescence microscope (Leica DM3000, Wetzlar, Germany). The activity of mitochondrial respiratory chain complexes (I–V) was detected using the Mouse Mitochondrial Respiratory Chain Complexes I–V ELISA Kit (Engreen Biosystem, Shanghai, China). The absorbance values were measured using a fluorescence microplate reader (same as above).

#### 4.1.6. Inflammation and Oxidative Stress Detection

Inflammatory cytokines (TNF-α, IL-1β, IL-6) in the cell culture supernatant were detected using ELISA kits (Beyotime, Shanghai, China). Intracellular ROS levels were measured using the DCFH-DA probe (MedChemExpress, Monmouth Junction, NJ, USA). The level of MDA was detected using an MDA Assay Kit (Beyotime, Shanghai, China). The absorbance values were measured using a fluorescence microplate reader (same as above).

#### 4.1.7. Western Blot Analysis

Total protein was extracted using lysis buffer (Thermo, Waltham, MA, USA) and quantified using a BCA Protein Assay Kit (Beyotime, Shanghai, China). Denatured proteins were separated by SDS-PAGE (GenScript, Piscataway, NJ, USA) and transferred to PVDF membranes (Millipore, Burlington, MA, USA). The membranes were blocked with 5% skim milk and incubated with primary antibodies against p38 MAPK, phosphorylated p38 MAPK, IκB alpha, and phosphorylated IκB alpha (Beyotime, Shanghai, China) at 4 °C. The membranes were then incubated with diluted secondary antibodies (Beyotime, Shanghai, China) (1:1000). Band intensities were quantified using ImageJ software (Version: 1.53t, National Institutes of Health, Bethesda, MD, USA).

### 4.2. Animal Experiments

#### 4.2.1. Animal Husbandry and Administration

Six-week-old male C57BL/6J mice weighing 21–23 g were selected for this study. The animals were acclimatized for one week before the experiment began. After acclimatization, the mice were randomly assigned to four groups (vehicle control group, BPAF-0.5, 4, 32 mg/kg), with six animals per group. All animals were administered BPAF corn oil solution (0, 0.1, 0.8, 6.4 mg/mL) by oral gavage at a volume of 5 mL/kg body weight, once daily. The study lasted for 90 days, after which mice from each group were sacrificed, and serum and liver tissues were collected for subsequent evaluation.

The animal housing conditions were strictly controlled to ensure a stable environment for the mice. The animals were housed in a temperature-controlled room maintained at (22 ± 2) °C with a humidity level of (50 ± 10)%, and a 12 h light/12 h dark cycle.

The study was approved by the Animal Ethics Committee of the Shanghai Municipal Center for Disease Control and Prevention (IACUC-PZ-2024-038) and conducted in accordance with the Guide for the Care and Use of Laboratory Animals published by the US National Institutes of Health.

Dose-selection rationale: the 90-day sub-chronic gavage study started at 0.5 mg kg^−1^, the lowest observed adverse-effect level (LOAEL) previously reported for BPAF-induced hepatotoxicity, and reached an upper limit of 32 mg kg^−1^, equivalent to 1/5 of the mouse LD_50_ (The data presented here are from preliminary experiments conducted by our research group and do not correspond to previously published work). Sample size (*n* = 6 per group) was calculated from a pilot ALT variance; with Power = 0.80 and α = 0.05 this number allows detection of a 30% difference in serum ALT using G*Power 3.1.9.7.

#### 4.2.2. Humane Endpoints and Animal Sacrifice

Humane endpoints were defined as significant weight loss (>20% of initial body weight), signs of severe distress (e.g., labored breathing, lethargy), or failure to thrive. Animals meeting these criteria were euthanized immediately to minimize suffering. Animals were euthanized by intraperitoneal injection of sodium pentobarbital (100 mg/kg) followed by cervical dislocation. All efforts were made to minimize suffering, including the use of analgesics and anesthetics where appropriate.

#### 4.2.3. Body Weight Measurement

Body weight was measured once per week during the experimental period.

#### 4.2.4. Liver Weight and Organ Index Measurement

At the end of the experiment, all animals were subjected to gross dissection, and the absolute weight of the liver was recorded. The relative weight (liver-to-body ratio) was calculated.

#### 4.2.5. Biochemical Analysis of Blood

Inflammatory cytokine levels in the serum were measured using ELISA: serum amyloid A (SAA) (Mouse SAA, BioVendor #RD291001200R, Asheville, NC, USA), TNF-α, IL-1β, IL-6 (the same Beyotime ELISA kit, Shanghai, China). The absorbance values were measured using a fluorescence microplate reader (same as above).

#### 4.2.6. Hematoxylin and Eosin (H&E) Staining

Liver tissues from mice with BPAF-induced chronic liver injury were fixed in 4% paraformaldehyde (Senbeiga, Nanjing, China) overnight, dehydrated in ethanol, and embedded in paraffin. Paraffin blocks were sectioned at 5 µm and stained with hematoxylin (Solarbio, Beijing, China) and eosin (Sangon, Shanghai, China). Sections were observed and photographed under a light microscope (Leica DM3000, Wetzlar, Germany).

#### 4.2.7. Immunofluorescence Staining of Frozen Sections

Liver tissue samples from mice with BPAF-induced chronic liver injury were prepared as frozen sections. Sections were fixed in 4% paraformaldehyde (Senbeiga, Nanjing, China) at room temperature for 30–60 min, washed three times with PBS (5 min each), treated with 0.3% Triton X-100 (Sigma-Aldrich, St. Louis, MO, USA) (in PBS) at room temperature for 15 min, and washed three times with PBS (5 min each). Sections were blocked with immunostaining blocking solution (Beyotime, Shanghai, China) at 37 °C for 2 h (no need to wash). Primary antibody: NF-κB p65 Rabbit Monoclonal Antibody (Beyotime, Shanghai, China) was diluted in immunostaining antibody dilution solution (Beyotime, Shanghai, China). Tissues were circled with a blocking pen and incubated with a small amount of antibody in a humidified chamber at 4 °C overnight. Sections were washed three times with PBS (5 min each) and incubated with an anti-rabbit FITC immunofluorescence staining kit (Beyotime, Shanghai, China) in the dark at 37 °C for 2 h. Sections were washed three times with PBS (5 min each) and stained with 2 µg/mL DAPI (Sewell, Yangxin, China) for nuclei. Sections were washed three times with PBS (5 min each) and mounted with an anti-fade mounting medium. For images comparing fluorescence intensity, the acquisition parameters of the fluorescence microscope must be kept consistent, and fluorescence intensity was analyzed using ImageJ software.

#### 4.2.8. Fluorescence Microplate Reader Analysis of M1 and M2 Macrophage Ratios

Due to the absence of flow cytometry, we used a split-sample plate-reader assay which yields bulk fluorescence and lacks single-cell resolution; thus data are presented as ‘polarization trend’ rather than absolute subset frequency. Single-cell suspension preparation was the same as described in Section 2.2.7. Because a flow cytometer was not available in our laboratory at the time, each liver-derived sample was divided into two equal aliquots (500 μL each); one aliquot was incubated with the M1 antibody cocktail (F4/80 + CD11c) and the other with the M2 antibody cocktail (F4/80 + CD206), followed by staining with an FITC-conjugated secondary antibody. Fluorescence intensity was measured on a Tecan SPARK microplate reader (Ex/Em 485/535 nm, Tecan, Canberra, Australia) and is expressed as relative fluorescence units (RFU). This approach provides only bulk signal and lacks single-cell resolution.

#### 4.2.9. Transmission Electron Microscopy

Tissue samples were collected and fixed in electron microscopy fixative (Servicebio, Wuhan, China) in the dark at room temperature for 2 h, dehydrated with acetone, and polymerized in an oven at 60 °C for 48 h. Ultrathin sections (60–80 nm) were cut and stained with 2% saturated uranyl acetate solution and 2.6% lead citrate solution. Mitochondrial morphological changes were observed under a transmission electron microscope (HT7700 Exalens, Hitachi, Tokyo, Japan).

### 4.3. Statistical Analysis

Data were analyzed using GraphPad Prism 8 and presented as means ± standard error (SE). To assess differences among multiple groups, one-way ANOVA was employed. The F-value is used to test whether the variances among different groups are significantly different. A higher F-value indicates a greater likelihood that the differences observed among the groups are not due to random chance. The *p*-value, on the other hand, represents the probability that the observed differences among the groups are statistically significant. Specifically, a *p*-value less than 0.05 is commonly used as a threshold to determine statistical significance. When the *p*-value is below this threshold, it suggests that the differences between the groups are unlikely to have occurred by chance alone, and thus, the group differences are considered statistically significant. Differences in RFU were analyzed using Dunnett’s test.

One-way ANOVA followed by Tukey’s or Dunnett’s post hoc test was used. *p* < 0.05 was considered statistically significant. This post hoc test helps to identify which specific groups differ from each other while controlling for the family-wise error rate. Significance was set at *p* < 0.05. Sample size was *n* = 6 per group. One-way ANOVA thus had between-group df = 3 and within-group (error) df = 20. All Dunnett’s or Tukey’s post hoc comparisons used these same error degrees of freedom.

## 5. Conclusions

This study demonstrates that VDAC1 knockdown alleviates Bisphenol AF-induced succinate accumulation, mitochondrial dysfunction, and inflammatory cytokine release in RAW264.7 macrophages. These protective effects are mediated, at least in part, via suppression of the p38 MAPK/NF-κB axis. Our findings identify VDAC1 as a potential therapeutic target for mitigating metabolic and inflammatory toxicity associated with bisphenol AF exposure.

## Figures and Tables

**Table 1:** The list of primers for qRT-PCR.

Gene Name	Forward Primer	Reverse Primer
*Vdac1*	AGTGACCCAGAGCAACTTCGCA	CAGGCGAGATTGACAGCAGTCT
β-actin	CATTGCTGACAGGATGCAGAAGG	TGCTGGAAGGTGGACAGTGAGG

## Data Availability

The datasets generated and analyzed during this study are available in the National Genomics Data Center (NGDC) repository under accession number OMIX010174. Additional data supporting the findings of this study are included in this article and its Supplementary Materials. Requests for further information should be directed to the corresponding author.

## Supplementary Materials

The following supporting information can be downloaded at: https://www.mdpi.com/article/10.3390/ph18111600/s1, Figure S1: Necrotic area quantification in liver sections; Table S1: Statistical summary of key endpoints (one-way ANOVA + Dunnett’s test); Table S2: Serum biochemical parameters of mice after 90-day BPAF exposure (mean ± SD, n = 6); Table S3: Blind histopathological evaluation of liver sections (Ishak scoring system, 0–18); Table S4: Relative M1- or M2-associated fluorescence intensity (RFU).
