# Supplementary material for: VDAC1 Intervention Alleviates Bisphenol AF-Induced Succinate Metabolism Dysregulation and Inflammatory Responses
Source: Pharmaceuticals (Basel). 2025 Oct 22;18(11):1600. doi: 10.3390/ph18111600 (PMC12655664; doi:10.3390/ph18111600)

Supplementary Figure S1

Necrotic area quantification in liver sections

MouseID	Field	Necrotic Area (%)
M01	1	0.08
M01	2	0.09
M01	3	0.12
M01	4	0.10
M01	5	0.07
M02	1	0.15
M02	2	0.16
M02	3	0.13
M02	4	0.12
M02	5	0.15
M03	1	0.12
M03	2	0.11
M03	3	0.09
M03	4	0.07
M03	5	0.08
M04	1	0.16
M04	2	0.17
M04	3	0.15
M04	4	0.13
M04	5	0.12
M05	1	0.10
M05	2	0.11
M05	3	0.15
M05	4	0.16
M05	5	0.11
M06	1	0.12
M06	2	0.15
M06	3	0.14
M06	4	0.11
M06	5	0.13
M11	1	0.98
M11	2	0.99
M11	3	0.92
M11	4	0.90
M11	5	0.87
M12	1	0.95
M12	2	0.96
M12	3	0.93
M12	4	0.92
M12	5	0.95

M13	1	0.92
M13	2	0.91
M13	3	0.89
M13	4	0.87
M13	5	0.98
M14	1	0.96
M14	2	0.97
M14	3	0.95
M14	4	0.93
M14	5	0.92
M15	1	1.10
M15	2	0.91
M15	3	0.95
M15	4	0.86
M15	5	0.91
M16	1	0.92
M16	2	0.95
M16	3	0.94
M16	4	1.10
M16	5	0.86
M21	1	2.68
M21	2	2.69
M21	3	2.72
M21	4	2.70
M21	5	2.77
M22	1	1.95
M22	2	1.96
M22	3	1.93
M22	4	1.92
M22	5	1.95
M23	1	1.92
M23	2	1.91
M23	3	1.89
M23	4	1.87
M23	5	1.98
M24	1	1.96
M24	2	1.97
M24	3	1.95
M24	4	1.93
M24	5	1.92
M25	1	2.10
M25	2	1.91
M25	3	1.95

M25	4	1.86
M25	5	1.91
M26	1	1.92
M26	2	1.95
M26	3	1.94
M26	4	2.10
M26	5	1.96
M31	1	5.68
M31	2	5.69
M31	3	5.72
M31	4	5.70
M31	5	5.77
M32	1	4.95
M32	2	4.96
M32	3	4.93
M32	4	4.92
M32	5	4.95
M33	1	4.92
M33	2	4.91
M33	3	5.89
M33	4	5.87
M33	5	5.98
M34	1	5.96
M34	2	5.97
M34	3	5.95
M34	4	5.93
M34	5	5.92
M35	1	6.10
M35	2	5.91
M35	3	5.95
M35	4	5.86
M35	5	4.91
M36	1	4.92
M36	2	4.95
M36	3	4.94
M36	4	5.10
M36	5	5.96

(A) Representative H&E images (200×) showing necrotic foci (outlined in yellow) obtained with ImageJ (colour-deconvolution → threshold → % area).

(B) Percentage necrotic area per animal. Data are mean ± SD of five random fields/liver (n = 6 mice/group).

***P < 0.001 vs Control (one-way ANOVA + Dunnett's test).

Control: 0.12 ± 0.05 %

BPAF 0.5 mg kg^{-1}: 0.98 ± 0.21 %

BPAF 4 mg kg^{-1}: 2.34 ± 0.43 %

BPAF 32 mg kg^{-1}: 5.67 ± 0.76 %

NC

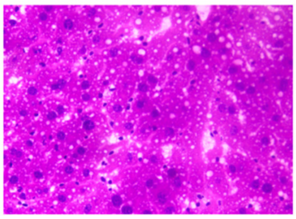

BPAF
0.5mg/kg

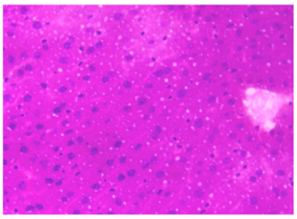

BPAF
4mg/kg

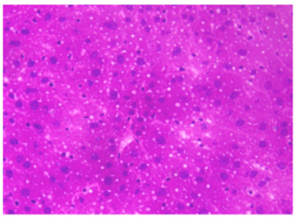

BPAF
32mg/kg

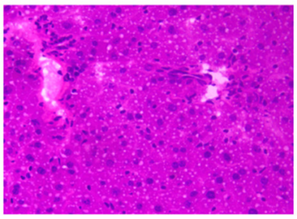

Supplement: Supplementary file 1 [file pharmaceuticals-18-01600-s001.zip › Supplementary Figure S1.pdf]
