# Supplementary material for: VDAC1 Intervention Alleviates Bisphenol AF-Induced Succinate Metabolism Dysregulation and Inflammatory Responses
Source: Pharmaceuticals (Basel). 2025 Oct 22;18(11):1600. doi: 10.3390/ph18111600 (PMC12655664; doi:10.3390/ph18111600)
Supplement: Supplementary file 1 [file pharmaceuticals-18-01600-s001.zip › Table S1 Statistical summary of key endpoints (one-way ANOVA + Dunnett’s test).pdf]

Table S1 Statistical summary of key endpoints (one-way ANOVA + Dunnett's test)

Endpoint	Fig./Tab.	F (3,20)	P-value	η^2 (effect size)	Dunnett's vs Ctrl P<0.05
Serum ALT	Tab S3	18.7	<0.001	0.74	4 & 32 mg kg^{-1}
Serum AST	Tab S3	21.4	<0.001	0.76	4 & 32 mg kg^{-1}
Serum BUN	Tab S3	6.5	0.003	0.49	32 mg kg^{-1}
Succinate (2500 nM)	Fig 1B	15.3	<0.001	0.70	500 & 2500 nM
SDH activity	Fig 1E	19.8	<0.001	0.75	≥100 nM
TNF-α (2500 nM)	Fig 2A	22.6	<0.001	0.77	≥100 nM
IL-6 (2500 nM)	Fig 2A	28.1	<0.001	0.81	≥100 nM
Ishak total score	Tab S2	14.9	<0.001	0.69	4 & 32 mg kg^{-1}
Necrotic area %	Fig S2	17.2	<0.001	0.72	4 & 32 mg kg^{-1}
M1 RFU (32 mg kg^{-1})	Fig 6B	8.3	0.001	0.55	32 mg kg^{-1}
M2 RFU (32 mg kg^{-1})	Fig 6B	2.1	0.133	0.24	—

η^2 (eta-squared) interpreted: 0.14 medium, 0.26 large effect.
