# Supplementary material for: VDAC1 Intervention Alleviates Bisphenol AF-Induced Succinate Metabolism Dysregulation and Inflammatory Responses
Source: Pharmaceuticals (Basel). 2025 Oct 22;18(11):1600. doi: 10.3390/ph18111600 (PMC12655664; doi:10.3390/ph18111600)
Supplement: Supplementary file 1 [file pharmaceuticals-18-01600-s001.zip › Table S2 Blind histopathological evaluation of liver sections (Ishak scoring system, 0–18).pdf]

Table S2. Blind histopathological evaluation of liver sections (Ishak scoring system, 0–18)

**Group**	**Animal ID**	**Interface & lobular inflammation**	**Portal inflammation**	**Fibrosis score**	**Total Ishak score**
Control	M01	0	0	0	0
	M02	0	0	0	0
	M03	1	0	0	1
	M04	0	0	0	0
	M05	0	0	0	0
	M06	0	0	0	0
	Mean ± SD	0.17 ± 0.41	0 ± 0	0 ± 0	0.17 ± 0.41
BPAF 0.5 mg kg^{-1}	M07	1	1	0	2
	M08	0	1	0	1
	M09	1	0	0	1
	M10	1	1	0	2
	M11	0	0	0	0
	M12	1	1	0	2
	Mean ± SD	0.67 ± 0.52	0.50 ± 0.55	0 ± 0	1.17 ± 0.75
BPAF 4 mg kg^{-1}	M13	1	1	0	2
	M14	2	1	0	3
	M15	1	2	0	3
	M16	1	1	0	2
	M17	2	1	0	3
	M18	1	1	0	2
	Mean ± SD	1.33 ± 0.52	1.17 ± 0.41	0 ± 0	2.50 ± 0.55 **
BPAF 32 mg kg^{-1}	M19	2	2	1	5
	M20	2	3	1	6

Group	Animal ID	Interface & lobular inflammation	Portal inflammation	Fibrosis score	Total Ishak score
	M21	3	2	1	6
	M22	2	2	0	4
	M23	3	3	1	7
	M24	2	2	1	5
	Mean ± SD	2.33 ± 0.52 ###	2.33 ± 0.52 ###	0.83 ± 0.41	5.50 ± 0.84 ###

** $P < 0.01$, ### $P < 0.001$ vs Control (Kruskal-Wallis followed by Dunn's test).
