# Supplementary material for: VDAC1 Intervention Alleviates Bisphenol AF-Induced Succinate Metabolism Dysregulation and Inflammatory Responses
Source: Pharmaceuticals (Basel). 2025 Oct 22;18(11):1600. doi: 10.3390/ph18111600 (PMC12655664; doi:10.3390/ph18111600)
Supplement: Supplementary file 1 [file pharmaceuticals-18-01600-s001.zip › Table S3 Serum biochemical parameters of mice after 90-day BPAF exposure (mean ± SD, n = 6).pdf]

Table S3 Serum biochemical parameters of mice after 90-day BPAF exposure (mean ± SD, n = 6)

Group	ALT ($U\ L^{-1}$)	AST ($U\ L^{-1}$)	CREA ($mg\ dL^{-1}$)	BUN ($mg\ dL^{-1}$)
Control	28.4 ± 3.1	71.5 ± 6.4	0.32 ± 0.04	18.7 ± 1.9
BPAF 0.5 $mg\ kg^{-1}$	31.2 ± 4.5	76.8 ± 7.2	0.33 ± 0.05	19.4 ± 2.3
BPAF 4 $mg\ kg^{-1}$	38.6 ± 5.7*	89.3 ± 8.1**	0.35 ± 0.04	20.8 ± 2.1
BPAF 32 $mg\ kg^{-1}$	47.9 ± 6.8***	105.7 ± 9.6***	0.38 ± 0.06*	23.5 ± 3.0**

*$P < 0.05$, **$P < 0.01$, ***$P < 0.001$ vs Control (one-way ANOVA followed by Dunnett’s test).
